# Impact of *Tetragenococcus halophilus* CICC 10286 Inoculation on the Fermentation Dynamics of Soybean Paste

**DOI:** 10.3390/foods15101744

**Published:** 2026-05-15

**Authors:** Jing Cai, Ling Zhang, Hao Zhou, Xingjiang Li, Shaotong Jiang

**Affiliations:** 1 National Engineering Research Center for Fermentation Technology, School of Food and Biological Engineering, Hefei University of Technology, No. 420 Feicui Road, Hefei 230601, China; 2 School of Biological and Food Engineering, Suzhou University, No. 49 Bianhe Central Road, Suzhou 234000, China

**Keywords:** high-salt fermented food, halophilic bacteria, fortified fermentation, amino acid profile, microbial succession

## Abstract

Fermented soybean paste, a traditional high-salt condiment, faces challenges in standardization and quality control due to its reliance on natural fermentation. This study systematically evaluated the effects of a defined starter culture, *Tetragenococcus halophilus* CICC 10286, on soybean paste fermentation by comparing natural fermentation (NF) and fortified fermentation (FF). Compared with NF, FF maintained a higher moisture in the later stage (NF-LS: 50.30%; FF-LS: 60.08%) and lower total acid levels in the middle and later stages (NF-MS: 1.58 g/100 g; FF-MS: 0.96 g/100 g; NF-LS: 2.23 g/100 g; FF-LS: 1.11 g/100 g). Although protein degradation was more pronounced in the FF group at the midpoint (*p* < 0.0001), the lower accumulation of amino acid nitrogen suggests a potential shift in nitrogen metabolism, possibly toward enhanced transamination or deamination processes. Free amino acid profiling indicated that FF facilitated earlier accumulation of umami and sweet amino acids, but the total free amino acid content in the later stage was lower. Specifically, Glu and Asp reached 724.47 nmol/L and 305.52 nmol/L, respectively, in NF-LS, whereas the corresponding values in FF-LS were 397.16 nmol/L and 275.46 nmol/L. Meanwhile, Pro reached 337.81 nmol/L in FF-MS, indicating earlier accumulation of some amino acids under FF. Notably, the proportion of bitter amino acids in the FF group was reduced in the later stage. Microbial community analysis showed that FF promoted the enrichment of *Tetragenococcus* and halotolerant bacteria, such as *Halomonas*, at the midpoint, and increased the relative abundance of the aroma-producing yeast *Zygosaccharomyces* (NF-MS: 37.73%; FF-MS: 65.11%). Functional prediction based on PICRUSt2 suggested a higher predicted abundance of genes involved in pyruvate metabolism and branched-chain amino acid degradation in the FF group. These findings demonstrate that *T. halophilus* CICC 10286, as a starter culture, can effectively modulate the fermentation of soybean paste, providing a scientific basis for developing standardized and quality-controlled fermentation processes.

## 1. Introduction

Fermented soybean paste is a staple condiment deeply rooted in East Asian culinary traditions, valued for its complex umami character, nutritional enrichment, and potential bioactive properties [1,2]. Its production has traditionally depended on natural fermentation, wherein the microbial community is shaped by indigenous microorganisms originating from raw materials and the production environment [3]. While this traditional approach yields products with distinctive regional flavor profiles, it poses inherent challenges for modern food manufacturing, including extended fermentation cycles, inconsistent batch-to-batch quality, potential safety risks associated with uncontrolled microbial succession, and limited scalability for industrial production [4]. Consequently, the development and application of defined starter cultures have become central strategies to achieve standardization, accelerate fermentation, enhance quality reproducibility, and ensure microbiological safety [5].

The biodiversity of bacterial and fungal species in soy sauce fermentation and recent technological advances, including the enhancement of flavor by starter cultures, have been extensively documented [6]. Among candidate starter microorganisms, *Tetragenococcus halophilus* has received considerable attention due to its ecological and metabolic adaptation to high-salt fermented foods. This moderately halophilic lactic acid bacterium has been consistently isolated from traditional high-salt fermented foods, including soy sauce, fish sauce, and salted seafood [7], and demonstrates remarkable osmoadaptation, thriving in NaCl concentrations up to 25% [8]. Beyond its stress tolerance, its functional contributions to fermentation ecosystems are substantial: it participates in amino acid conversion, shaping the volatile compound landscape and enhancing umami, sweetness, and overall taste complexity [9]. As an early colonizer, it may also influence microbial succession, stabilize community structure, and suppress undesirable microorganisms through competitive exclusion or bacteriocin-like activity [10].

Despite this substantial body of work, critical gaps remain in our understanding of *T. halophilus* application in soybean paste fermentation. Prior studies on *T. halophilus* inoculation have typically assessed either end-point quality parameters or microbial community composition, but rarely both in an integrated, time-resolved manner [11]. Consequently, key questions still need to be addressed [12,13,14]: How does inoculation with *T. halophilus* reshape microbial community succession dynamics? Does it enhance aminogenesis and improve the amino acid profile? How does its introduction alter the potential metabolism of the fermentation microbiota? Answering these questions is crucial for developing an effective, defined starter strategy for soybean paste fermentation [15].

To address this gap, this study evaluated the fermentation of soybean paste using both traditional natural methods and a process fortified with *T. halophilus* CICC 10286. Through an integrated analysis of physicochemical properties, amino acid profiles, and the microbial community, the impact of using this defined halophilic starter was elucidated. These findings provide a robust scientific basis for designing optimized starter cultures and controlled fermentation protocols, which can advance the standardization, quality, and innovation of traditional fermented soybean products for the global food industry.

## 2. Materials and Methods

### 2.1. Sample Collection

The samples used in this study were collected from a soybean paste manufacturer in Anhui Province, China. For the natural fermentation method, the soybeans (*Glycine max*) were steamed and then mixed with wheat flour (*Triticum aestivum* L.) in a 3:1 (*w*/*w*) ratio. Thereafter, water (2:1, *w*/*w*), salt (5%, *w*/*v*), sugar (10%, *w*/*v*), and licorice (5%, *w*/*v*) were added to the mixture in a vat. The soybean paste was obtained after six months of outdoor natural fermentation without temperature control. Based on the natural fermentation (NF), sampling was conducted at three key time points: the early (ES, 0 months), midpoint (MS, 3 months), and later (LS, 6 months) stages of the fermentation period. At each stage, triplicate samples were collected to ensure the representativeness and reliability of the data. For the fortified fermentation method, *T. halophilus* CICC 10286 was selected as the starter culture. *T. halophilus* CICC 10286 was obtained from the China Center of Industrial Culture Collection (CICC), which is specifically used in the fermentation of soybean products. The strain was activated by inoculating 0.5 mL of the bacterial suspension into a test tube containing 5 mL of MRS broth (Guangdong Huankai Microbial Science & Technology Co., Ltd., Guangzhou, China), followed by incubation at 37 °C for 24 h. Then, 1 mL of the activated culture was transferred into 100 mL of fresh MRS broth and incubated at 37 °C for another 24 h to prepare the seed culture for fermentation. Before fermentation began, 20 μL of seed culture was diluted with ultrapure water and sprayed onto each 100 g of raw material. Similarly, samples from the fortified fermentation (FF) were collected at the same three time points, with three replicates per time point.

Each sample was divided into two portions: one was stored at 4 °C for physicochemical and amino acids analysis, and the other was preserved at −80 °C for subsequent microbial DNA extraction and high-throughput sequencing.

### 2.2. Physicochemical and Amino Acids Analysis

Fermentation parameters are known to significantly influence the soy fermentation systems [16]. Moisture (%) was determined using the direct drying method according to the national standard GB 5009.3-2016. Samples were dried at 105 °C ± 2 °C to a constant weight, and the moisture content was calculated based on the weight loss [17]. Total acid (g/100 g) was measured using the acid-base indicator titration method described in GB 12456-2021. The sample solution was titrated with a standard NaOH solution (Sinopharm Chemical Reagent Co., Ltd., Shanghai, China) to the endpoint, and the total acidity was calculated from the volume of NaOH consumed [17]. Reducing sugar (%) was analyzed by the 3,5-dinitrosalicylic acid (DNS) colorimetric method. After extraction, the sample reacted with the DNS reagent (Shanghai Aladdin Biochemical Technology Co., Ltd., Shanghai, China). The mixture was heated under alkaline conditions to develop color, and the absorbance was measured at 540 nm. The content was calculated using a standard glucose calibration curve [18]. Protein (g/100 g) was determined following the national standard GB-5009.5-2025 using the Kjeldahl method [19]. The total nitrogen content was obtained and converted to protein content using the appropriate conversion factor. Amino acid nitrogen (g/100 g) was analyzed according to GB 5009.235-2016 using a pH meter method. After sample pretreatment, formaldehyde solution (Sinopharm Chemical Reagent Co., Ltd., Shanghai, China) was added to fix the amino groups, followed by titration with a standard NaOH solution to the endpoint. The amino acid nitrogen content was calculated based on the volume of NaOH used [17]. Free amino acids were analyzed using an automated amino acid analyzer. After extraction, centrifugation, and filtration, the pretreated sample was injected into the analyzer for the accurate determination of individual free amino acid contents [17].

### 2.3. Amplicon Sequencing and Processing

Total genomic DNA was extracted from the samples using the DNeasy PowerSoil Kit (QIAGEN China (Shanghai) Co., Ltd., Shanghai, China) following the manufacturer’s instructions. For bacterial community analysis, the V3-V4 hypervariable regions of the 16S rRNA gene were amplified with the universal primers 338F and 806R. For fungal community analysis, the internal transcribed spacer (ITS) region was amplified using the primers ITS1F and ITS2R [20].

The resulting PCR (Polymerase Chain Reaction) products were purified with the AxyPrep DNA Gel Extraction Kit (Axygen Biotechnology (Hangzhou) Limited, Hangzhou, China). The quality and concentration of the purified products were assessed by 2% agarose gel electrophoresis and quantified using the Quantus^TM^ Fluorometer (Promega (Beijing) Biotech Co., Ltd., Beijing, China) with a blue fluorescence quantification system. Sequencing libraries were constructed with the NEXTFLEX Rapid DNA-Seq Kit (Beijing Biootech Scientific Co., Ltd., Beijing, China) according to the following steps: (1) adapter ligation; (2) removal of self-ligated adapter fragments using bead-based purification; (3) library enrichment by limited-cycle PCR; and (4) purification of the final PCR products with beads to obtain the finished libraries. Paired-end sequencing (2 × 300 bp) was performed on the Illumina MiSeq platform.

Raw sequencing data were processed using the QIIME 2 2023.7 pipeline [21]. Paired-end reads were merged, and sequences were trimmed based on barcodes and primer lengths. Operational taxonomic units (OTUs) were clustered at 97% sequence similarity. Representative sequences from each OTU were taxonomically annotated against the SILVA database [22].

### 2.4. Statistical Analysis and Visualization

GraphPad Prism 10.4.1 was used for basic statistical analysis of physicochemical properties and alpha-diversity indices, and for generating corresponding graphs. Visualization of physicochemical parameters, alpha-diversity, community composition at the phylum and genus levels, and free amino acid profiles was also conducted using this software. Results are presented as mean ± standard deviation (SD) from three replicates, as indicated in the figure legends.

Multivariate statistical analyses were carried out in R (version 4.3.0). The Bray–Curtis distance matrix, Principal Coordinates Analysis (PCoA), and permutational multivariate analysis of variance (PERMANOVA) were all conducted using the vegan package [23]. Detrended Correspondence Analysis (DCA), distance-based redundancy analysis (db-RDA), the envfit function for vector fitting, and variation partitioning were also implemented with vegan. Hierarchical partitioning of the variation explained by physicochemical properties was performed using the rdacca.hp package [24]. The relationships and contributions of physicochemical properties were visualized via UpSet plots generated with the UpSetR package [25].

To identify differentially abundant microbial taxa, linear discriminant analysis effect size (LEfSe) was performed with the microeco package [26], using a significance threshold of *p* < 0.05 and a linear discriminant analysis (LDA) score > 2.0. Spearman correlation coefficients were calculated using the Hmisc package [27]. Correlation networks were constructed by filtering pairwise correlations with |r| ≥ 0.60 and *p* < 0.05. Network topology parameters were extracted using the igraph package [28], and the final correlation network was visualized in Gephi 0.10.1. Data wrangling and graphical visualization for the above analyses were accomplished using the tidyverse, ggplot2, and ggrepel packages.

Functional prediction using PICRUSt2 (Phylogenetic Investigation of Communities by Reconstruction of Unobserved States, version 2) was performed according to the official documentation [29]. Figures related to functional prediction were visualized and finalized using GraphPad Prism 10.4.1 and Origin 2025b.

## 3. Results and Discussion

### 3.1. Changes in Physicochemical Properties

Physicochemical properties are important parameters reflecting the quality of fermented soybean paste. Distinct dynamic patterns in these properties were observed between NF and FF across different fermentation stages. Two-way ANOVA results (Table 1) revealed that the fermentation method, the fermentation time, and their interaction all had significant effects on key physicochemical parameters during fermentation (*p* < 0.05).

**Figure 1:**
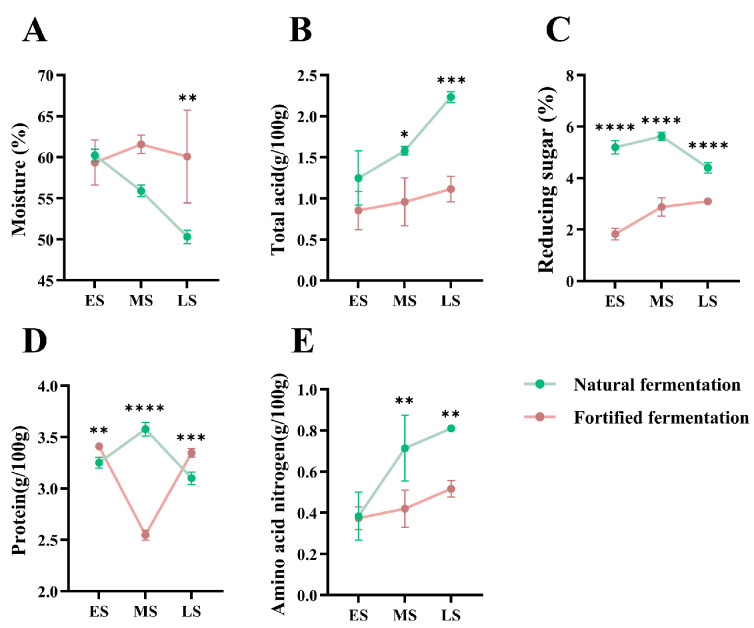
Changes in physicochemical properties during natural fermentation and fortified fermentation, including moisture (**A**), total acid (**B**), reducing sugar (**C**), protein (**D**), and amino acid nitrogen (**E**). ES, MS, and LS indicate the early, midpoint, and later stages, respectively. *, *p* < 0.05; **, *p* < 0.01; ***, *p* < 0.001; ****, *p* < 0.0001.

Moisture (Figure 1A) in the NF group decreased continuously from 59.50% to 50.30%, likely due to moisture migration driven by osmotic stress under high-salt conditions, whereas in the FF group it remained relatively stable, possibly because the modified fermentation process reduced osmotic stress and better retained water (*p* < 0.01). Total acid (Figure 1B) increased in both groups, accumulating faster in NF, which may be attributed to enhanced microbial acid production under traditional fermentation conditions (*p* < 0.05 at midpoint; *p* < 0.001 at later). Reducing sugar (Figure 1C) in NF initially rose then fell, reflecting dynamic starch hydrolysis followed by microbial consumption, while in FF it gradually accumulated, suggesting slower sugar utilization in the fortified system (*p* < 0.0001).

Protein degradation and amino acid nitrogen accumulation are crucial for flavor development [30]. Compared to other parameters, protein showed clear stage-specific differences (Figure 1D). Notably, in FF at the midpoint, protein decreased sharply (*p* < 0.0001), likely due to enhanced proteolytic enzyme activity under modified conditions. Although amino acid nitrogen increased in both groups, NF showed significantly higher levels at both the midpoint and later (Figure 1E). Interestingly, the pronounced protein degradation in FF at the midpoint did not correspond to proportionally higher amino acid nitrogen, indicating that fortified fermentation may alter nitrogen transformation pathways, possibly through differential protease action or metabolite conversion [31].

### 3.2. Free Amino Acid Composition

**Figure 2:**
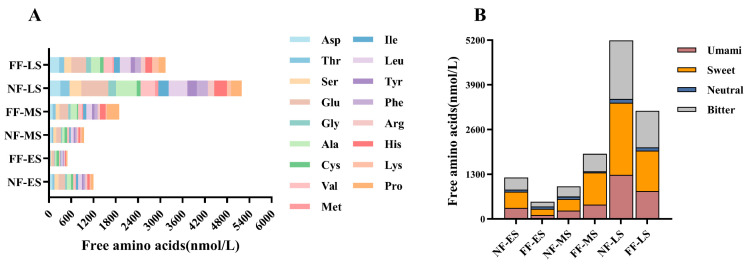
The composition of free amino acids based on individual type (**A**) and category according to taste characteristics (**B**). NF and FF indicate natural fermentation and fortified fermentation, respectively. ES, MS, and LS indicate the early, midpoint, and later stages, respectively.

Free amino acids, the products of protein degradation during soybean paste fermentation, serve as crucial taste contributors and important precursors for the formation of subsequent flavor compounds [32]. To further evaluate the effects of NF and FF on the accumulation of taste-active substances, the composition of free amino acids was analyzed at each stage. Overall, the total content in soybean paste increased markedly by the later stage under both methods. However, distinct accumulation patterns were observed: the NF group showed a fluctuating trend, whereas the FF group exhibited a continuous increasing trend (Figure 2).

Regarding individual free amino acids (Figure 2A), Glu (glutamic acid), Asp (aspartic acid), Ala (alanine), Leu (leucine), Val (valine), His (histidine), and Pro (proline) accounted for a high proportion in the later stage, indicating that the later stage is critical for the substantial release and accumulation of free amino acids. In particular, Glu and Asp in NF-LS reached 724.47 nmol/L and 305.52 nmol/L, respectively, which were notably higher than those in FF-LS (397.16 nmol/L and 275.46 nmol/L). Concurrently, amino acids such as Leu (505.13 nmol/L), Val (390.60 nmol/L), His (344.45 nmol/L), and Pro (286.37 nmol/L) also accumulated substantially in NF-LS. In contrast, the absolute contents of most free amino acids in the FF group in the later stage were lower than those in the NF group. However, the accumulation of some amino acids was more pronounced in the FF group at the midpoint; for instance, Pro in FF-MS reached 337.81 nmol/L, exceeding the level in NF-MS. The contents of His, Ala, and Val in FF-MS were also higher than those in NF-MS. These results suggest that FF promoted the earlier accumulation of certain amino acids at the midpoint, while attenuating their concentrated increase at the end.

Based on their taste characteristics, these free amino acids can be categorized into four groups [17]: umami amino acids (Asp, Glu), sweet amino acids (Thr, Ser, Gly, Ala, His, Val, Pro), neutral amino acids (Cys), and bitter amino acids (Met, Ile, Leu, Tyr, Phe, Lys, Arg). As summarized in Figure 2B, sweet amino acids consistently represented the most abundant category in all samples, followed by bitter amino acids, whereas umami and neutral amino acids were present at relatively lower levels. This indicates that the taste profile of soybean paste is not dominated by a single taste category but is collectively influenced by sweet, bitter, and umami amino acids, with sweet amino acids being predominant. A further comparison between the NF and FF groups revealed that FF was more conducive to the earlier accumulation of umami and sweet amino acids at the midpoint. However, in the later stage, the contents of umami, sweet, and bitter amino acids in NF-LS were all higher than those in FF-LS, with differences in sweet and bitter amino acids being particularly pronounced. Notably, although the umami amino acids accumulated to a higher level in the later stage of NF, this was accompanied by a substantial enrichment of bitter amino acids.

### 3.3. Microbial Diversity and Community Composition

**Figure 3:**
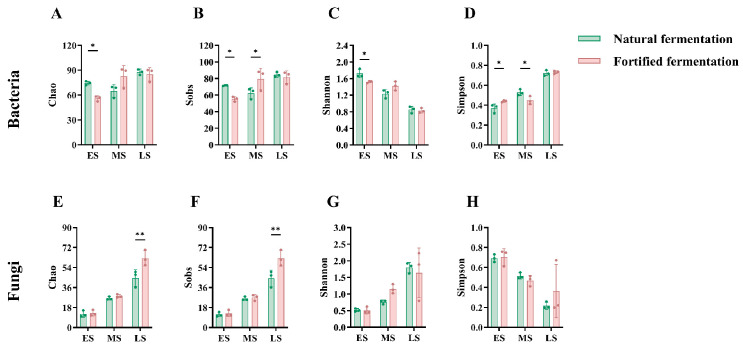
Alpha diversity during fermentation: (**A**–**D**) for bacteria and (**E**–**H**) for fungi. ES, MS, and LS indicate the early, midpoint, and later stages, respectively. *, *p* < 0.05; **, *p* < 0.01.

**Figure 4:**
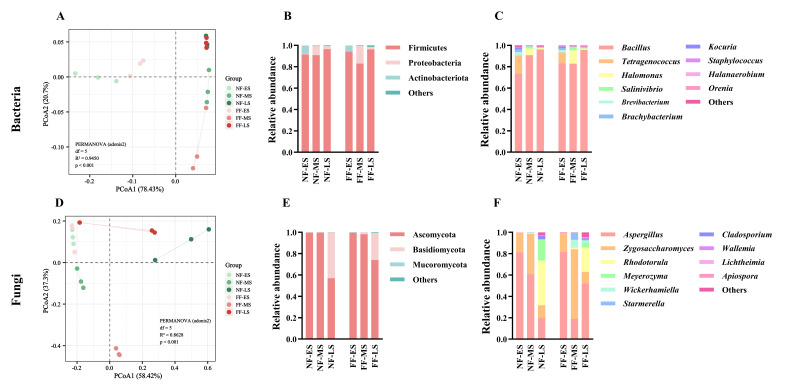
PCoA based on Bray–Curtis distance for bacteria (**A**) and fungi (**D**). Microbial composition at the phylum level for bacteria (**B**) and fungi (**E**). Microbial composition at the genus level for bacteria (**C**) and fungi (**F**). NF and FF indicate natural fermentation and fortified fermentation, respectively. ES, MS, and LS indicate the early, midpoint, and later stages, respectively.

In the alpha-diversity assessment (Figure 3), the Chao and Sobs indices primarily reflect species richness within a microbial community, while the Shannon and Simpson indices indicate species diversity and evenness, respectively [33]. Overall, bacterial communities exhibited higher Chao and Sobs indices than fungal communities, indicating greater species richness in bacteria during soybean paste fermentation. As fermentation progressed, the Chao (Figure 3A) and Sobs (Figure 3B) indices for bacteria displayed a fluctuating trend, initially decreasing and then increasing. In contrast, the Shannon (Figure 3C) index for bacteria declined continuously, while the corresponding Simpson (Figure 3D) index rose. For fungi, the trends across the four indices were more consistent: Chao1 (Figure 3E), Sobs (Figure 3F), and Shannon (Figure 3G) indices all increased steadily throughout fermentation, whereas the Simpson (Figure 3H) index decreased accordingly. Further comparison between fermentation methods at different stages revealed that the influence of FF on the bacterial community was mainly concentrated at the beginning and midpoint. Numerically, the alpha-diversity indices for the NF group were generally higher than those for the FF group. In contrast, the fungal community showed a weaker response to FF at the beginning and midpoint. However, in the later stage, the fungal species richness in the FF group was significantly higher than that in the NF group (*p* < 0.01), suggesting that FF primarily enhanced fungal species richness in the later stage. PCoA further demonstrated clear separation of sample groups for bacteria (Figure 4A) and fungi (Figure 4C), respectively. This indicates distinct microbial community structures for both bacteria and fungi across the different groups.

For bacteria, Firmicutes, Proteobacteria, and Actinobacteriota were the dominant phyla throughout fermentation. Among these, Firmicutes was absolutely dominant, with a relative abundance exceeding 80% in all samples (Figure 4C). At the genus level (Figure 4D), *Bacillus* was the most predominant genus, maintaining high relative abundance across all samples, confirming its role as a core bacterial genus under both fermentation methods. Besides *Bacillus*, *Tetragenococcus* also showed relatively high abundance in the early stage, reaching 17.08% in NF-ES and 10.30% in FF-ES, but its abundance declined rapidly thereafter, indicating its primary involvement in the initial fermentation phase. Notably, the average relative abundance of *Halomonas* in FF-MS was 12.51%, markedly higher than the 0.63% in NF-MS. Concurrently, *Salinivibrio* increased from 1.79% in NF-MS to 3.24% in FF-MS. These results suggest that FF promoted the enrichment of certain halotolerant bacterial genera during the midpoint, leading to a bacterial community structure distinct from that of the NF group.

For fungi, Ascomycota and Basidiomycota were the dominant phyla. Ascomycota was particularly dominant, accounting for nearly 100% of the relative abundance during the early and mid-point of fermentation. A significant structural shift occurred in the later stage, with the abundance of Basidiomycota rising rapidly (Figure 4E). At the genus level (Figure 4F), *Aspergillus* and *Zygosaccharomyces* were the dominant genera during the early and midpoint stages. In the early stage, *Aspergillus* dominated both NF and FF groups, with average relative abundances as high as 81.13% and 81.77%, respectively. During the midpoint, the relative abundance of *Zygosaccharomyces* in the FF group increased sharply to 65.11%, significantly surpassing the 37.73% in the NF group. *Zygosaccharomyces* is a halotolerant and osmotolerant aroma-producing yeast often associated with the formation of flavor compounds, such as alcohols and esters, in high-salt fermented foods [34]. By the later stage, both groups exhibited co-enrichment of multiple genera. However, *Rhodotorula* showed a higher average relative abundance in the NF group (41.79%), whereas *Aspergillus* regained dominance in the FF group (52.10%). Furthermore, genera such as *Cladosporium*, *Wallemia*, *Lichtheimia*, and *Apiospora* were detected at low proportions in the later stage. For instance, *Cladosporium* accounted for 3.14% in the NF group, while *Wallemia* and *Cladosporium* constituted 0.89% and 0.94%, respectively, in the FF group. Although their relative abundances were low, the presence of these genera indicates a more complex compositional profile within the fungal community during the later fermentation stage.

### 3.4. Differential Characteristics of Microbial Community

To identify the core microbes driving the succession differences between the two fermentation groups, LEfSe (*p* < 0.05, LDA > 2.0) was employed to identify significantly different biomarker taxa at each stage. Statistical results indicated that no statistically significant differential bacterial genera were detected in the later stage, and similarly, no significant differential fungal genera were found in the early stage. Significant species enrichment features were observed in all other fermentation periods.

**Figure 5:**
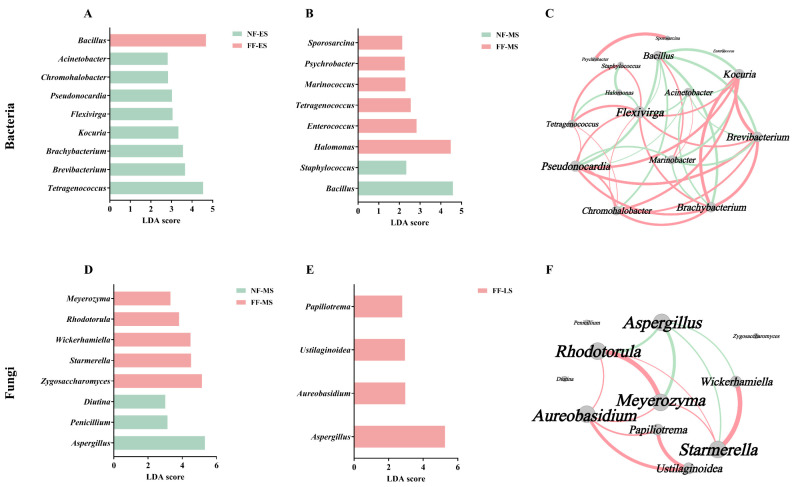
LEfSe analysis for bacteria at the early (**A**) and midpoint (**B**) stages, with the network based on differential bacteria (**C**). LEfSe analysis for fungi at the midpoint (**D**) and later (**E**) stages, with the network based on the differential fungi (**F**). NF and FF indicate natural fermentation and fortified fermentation, respectively. ES, MS, and LS indicate the early, midpoint, and later stages, respectively.

For bacterial communities, nine significantly differential genera were identified in the early stage (Figure 5A). Among them, *Bacillus* was significantly enriched in the FF group, exhibiting the highest LDA score (4.68), indicating it as the most representative differential dominant genus. In contrast, genera such as *Tetragenococcus*, *Brevibacterium*, and *Brachybacterium* were significantly enriched in the NF-ES group. In the midpoint stage, eight significantly differential bacterial genera were detected (Figure 5B). In the NF-MS group, only *Bacillus* and *Staphylococcus* were significantly enriched, with LDA scores of 4.58 and 2.33, respectively. Conversely, the number of differential genera increased in the FF-MS group. Notably, *Tetragenococcus*, which was significantly enriched in the NF group during the early stage, became significantly enriched in the FF group by the midpoint.

For fungal communities, eight significantly differential genera were identified in the midpoint stage (Figure 5D). Genera including *Aspergillus*, *Penicillium*, and *Diutina* were significantly enriched in the NF-MS group, with *Aspergillus* having the highest LDA score (5.32). In the FF-MS group, *Zygosaccharomyces*, *Starmerella*, *Wickerhamiella*, *Rhodotorula*, and *Meyerozyma* were significantly enriched, with *Zygosaccharomyces* showing the highest LDA score (5.14). This result indicates that *Aspergillus* remained the primary differential dominant genus in the NF group, whereas the FF group tended to enrich multiple yeast-like fungal genera. In the later stage, four significantly differential fungal genera were identified, all enriched in the FF group, with *Aspergillus* again exhibiting the highest LDA score (5.27) (Figure 5E).

To further explore the potential interactions among these differential microbial genera, correlation networks for bacteria (Figure 5C) and fungi (Figure 5F) were constructed based on the taxa identified by the LEfSe analysis. In the bacterial network, *Bacillus* (degree = 7) was a key hub node, showing significant negative correlations with several differential genera such as *Brevibacterium* and *Kocuria*, reflecting its dominant role in niche competition. *Tetragenococcus* (degree = 5) also displayed high connectivity, exhibiting significant positive correlations with genera like *Staphylococcus* and *Chromohalobacter*, suggesting potential synergistic relationships with some differential taxa in the high-salt fermentation environment. In the fungal network, *Aspergillus* (degree = 4) was one of the most connected nodes, showing significant negative correlations with yeasts such as *Rhodotorula*, *Meyerozyma*, and *Starmerella*. Notably, the aroma-producing yeast *Zygosaccharomyces* appeared isolated in the network, indicating no significant correlations with other differential fungal genera, which may suggest it occupies a relatively independent ecological niche.

### 3.5. Correlation Analysis Between Physicochemical Properties and Microorganisms

To further elucidate the influence of physicochemical properties on the structural changes in microbial communities during soybean paste fermentation, a db-RDA based on Bray–Curtis distance and hierarchical partitioning analysis were employed to analyze the relationships between the bacterial and fungal communities and the key physicochemical properties.

**Figure 6:**
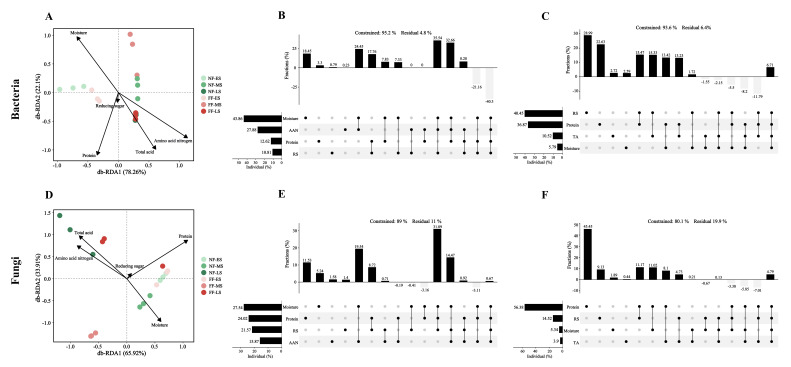
The db-RDA based on the Bray–Curtis distance between physicochemical properties and bacteria (**A**), using hierarchical partitioning analysis for natural fermentation (**B**) and fortified fermentation (**C**). Similarly, the db-RDA based on the Bray–Curtis distance between physicochemical properties and fungi (**D**), using hierarchical partitioning analysis for natural fermentation (**E**) and fortified fermentation (**F**). NF and FF indicate natural fermentation and fortified fermentation, respectively. ES, MS, and LS indicate the early, midpoint, and later stages, respectively. RS, reducing sugar; ANN, amino acid nitrogen; TA, total acid.

The db-RDA results revealed a strong overall association between the physicochemical properties and both bacteria (Figure 6A) and fungi (Figure 6D), with the overall model tests being statistically significant (*p* < 0.01). According to the envfit results, amino acid nitrogen (R^2^ = 0.45, *p* < 0.01), protein (R^2^ = 0.36, *p* < 0.05), and moisture (R^2^ = 0.34, *p* < 0.05) were the primary physicochemical factors significantly correlated with variations in the bacterial community structure. For the fungal community structure, protein (R^2^ = 0.64, *p* < 0.01) and total acid (R^2^ = 0.37, *p* < 0.05) were identified as the main physicochemical properties showing significant correlations.

Hierarchical partitioning analysis further delineated the differences in the driving physicochemical properties between the two fermentation groups. For the bacterial community, in the NF group, physicochemical properties collectively explained 95.2% of the total variation in community structure (Figure 6B). Moisture exhibited the highest independent explanatory proportion (46.07%), which was statistically significant (*p* < 0.05). In the FF group, the total variation explained by physicochemical properties was similar at 93.6% (Figure 6C). However, reducing sugar and protein showed the highest independent explanatory proportions (43.22% and 39.39%, respectively), both reaching significant levels (*p* < 0.01 and *p* < 0.05, respectively).

For the fungal community, in the NF group, physicochemical properties explained a total of 89.0% of the variation, slightly higher than in the FF group (Figure 6E). In the significance tests, none of the individual properties reached a significant level, although moisture (30.94%) and protein (26.99%) had relatively higher independent explanatory proportions. In contrast, within the FF group (Figure 6F), protein had the highest independent explanatory proportion (70.39%), and its significance test was significant (*p* < 0.01). This indicates that after fortified fermentation, the response of the fungal community to physicochemical changes became markedly concentrated on protein, establishing it as the key factor driving the structural shifts in the fungal community.

### 3.6. Metabolic Function Analysis Based on PICRUSt2

**Figure 7:**
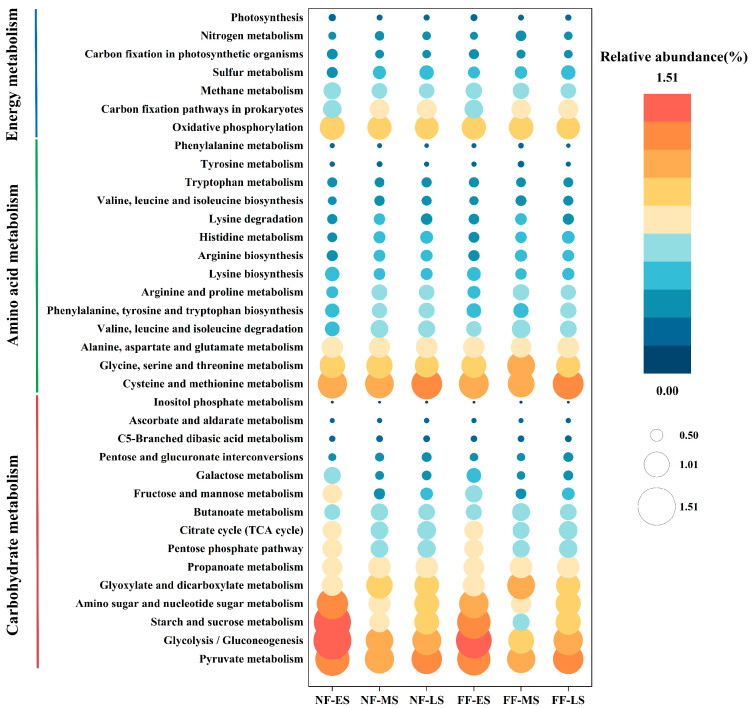
Functional prediction related to carbohydrate metabolism, amino acid metabolism, and energy metabolism based on PICRUSt2. NF and FF indicate natural fermentation and fortified fermentation, respectively. ES, MS, and LS indicate the early, midpoint, and later stages, respectively.

Based on the predicted functional potential from PICRUSt2, three KEGG level-2 pathways were selected as the most relevant to soybean paste fermentation [35]: carbohydrate metabolism, amino acid metabolism, and energy metabolism. The relative abundance changes in their respective level-3 pathways were further compared (Figure 7). Overall, these three functional categories maintained high predicted abundance across all samples, though variations in the relative abundance of certain level-3 pathways were observed depending on the fermentation stage and method.

Pathways related to carbohydrate metabolism, which are expected to provide crucial carbon sources for microbial growth and subsequent metabolism [36], exhibited relatively high predicted abundance, particularly in the early stages (Figure 7). Pathways such as glycolysis/gluconeogenesis, pyruvate metabolism, and starch and sucrose metabolism showed high abundance during the early stage. A comparative analysis revealed that the relative abundances of the starch and sucrose metabolism pathway in the FF-ES and FF-MS groups were slightly lower than those in their NF group counterparts. In contrast, pathways for pyruvate metabolism and glyoxylate and dicarboxylate metabolism showed higher predicted abundance in the FF-MS group, suggesting a potentially enhanced genetic potential for downstream carbon metabolism in the FF group during the midpoint.

Amino acid metabolism pathways are closely linked to the formation of umami substances and certain aroma precursors in soybean paste [37]. Among all samples, cysteine and methionine metabolism and glycine, serine and threonine metabolism consistently showed high predicted abundance. Further comparison indicated that the arginine and proline metabolism pathway had a slightly higher relative abundance in the FF-MS group than in the contemporaneous NF group, implying a potentially greater genetic potential related to proline in the FF group at the midpoint. Additionally, the valine, leucine and isoleucine degradation pathway showed higher predicted abundance in the FF group during the midpoint and later stages. This suggests a greater predicted potential for the conversion of branched-chain amino acids in the FF group, which aligns with the observed lower accumulation of bitter amino acids in the later stage of the FF group. These observed differences in the predicted abundance of specific amino acid metabolism pathways may be attributed to variations in fermentation conditions, which have been demonstrated in related fermentation systems [38].

Pathways associated with energy metabolism remained relatively stable overall. Among them, the oxidative phosphorylation pathway maintained a high predicted abundance across all groups, indicating a consistently stable potential for energy metabolism within the microbial communities throughout fermentation.

While our findings provide insights into how *T. halophilus* modulates the fermentation process, this work represents a foundational investigation. Consequently, the observed changes in key parameters are strong indicators of potential quality improvement, but they must be validated by descriptive sensory evaluation in subsequent application-focused studies. Furthermore, the critical aspect of food safety, particularly regarding biogenic amine formation, was not addressed experimentally here. Future work will include targeted analysis of biogenic amines and a safety assessment of the final product. These planned studies will bridge the gap between the process modulation demonstrated here and the comprehensive assessment of final product quality and safety required for standardized production.

## 4. Conclusions

The inoculation of *T. halophilus* as a defined starter culture influenced the fermentation pattern of soybean paste compared to natural fermentation. Specifically, it influenced microbial succession, contributing to the early-stage prevalence of halotolerant bacteria and favoring the growth of aroma-producing yeasts, such as *Zygosaccharomyces*, at the midpoint stage. These microbial shifts were associated with alterations in metabolic profiles, particularly in amino acid metabolism. This appeared to lead to an earlier accumulation of taste-active amino acids and a reduction in bitter amino acid content in the final product. Moreover, the fungal community in fortified fermentation exhibited a stronger correlation with protein degradation, highlighting the role of key physicochemical properties in shaping microbial ecosystems under these controlled conditions. Collectively, these findings suggest the potential of defined halophilic starters to guide fermentation processes and improve product consistency in traditional soybean paste production. Further research is needed to optimize starter formulations and fermentation parameters, which would be crucial for evaluating the practical industrial applicability of this approach for the standardization of high-salt fermented foods.

## Figures and Tables

**Table 1:** Two-way ANOVA results for the physicochemical properties.

	Fermentation Group			Fermentation Stage			Interaction		
	F	*p*	η^2^	F	*p*	η^2^	F	*p*	η^2^
Moisture	15.0900	0.0022	0.5570	4.9510	0.0270	0.4520	6.1790	0.0143	0.5070
Total acid	49.2000	<0.0001	0.8040	12.9400	0.0010	0.6830	4.4600	0.0356	0.4260
Reducing sugar	512.9000	<0.0001	0.9770	15.8800	0.0004	0.7260	31.4600	<0.0001	0.8400
Protein	76.6900	<0.0001	0.8650	43.2300	<0.0001	0.8780	301.3000	<0.0001	0.9800
Amino acid nitrogen	20.5700	0.0007	0.6320	14.5600	0.0006	0.7080	4.6370	0.0322	0.4360

## Data Availability

The original contributions presented in this study are included in the article. Further inquiries can be directed to the corresponding authors.
